# A nanoparticle-mist deposition method: fabrication of high-performance ITO flexible thin films under atmospheric conditions

**DOI:** 10.1038/s41598-021-90028-6

**Published:** 2021-05-19

**Authors:** Ryoko Suzuki, Yasutaka Nishi, Masaki Matsubara, Atsushi Muramatsu, Kiyoshi Kanie

**Affiliations:** 1 Institute of Multidisciplinary Research for Advanced Materials, Tohoku University, Sendai, 980-8577 Japan; 2 Nikon Corporation, 10-1, Asamizodai, 1-chome, Minami-ku, Sagamihara-City, 252-0328 Japan; 3 National Institute of Technology, Sendai College, 48 Nodayama, Medeshima-Shiote, Natori, 981-1239 Japan

**Keywords:** Materials chemistry, Materials for devices, Nanoscale materials

## Abstract

Indium tin oxide (ITO) thin films with low resistivity and high transparency in the visible light region have been prepared on flexible plastic films by a deposition method using water mist containing ITO nanoparticles (NPs) under atmospheric conditions. The ITO NP-mist was generated by ultrasonic irradiation of a water dispersion. Our developed protrusion-rich ITO NPs were applied as the ITO NPs. The ITO NPs show high dispersion stability in water without the use of any dispersant. Comparison investigations revealed that utilization of the ITO NPs played a critical role in fabricating high-performance ITO thin films on flexible films, and the resistivity reached 9.0 × 10^−3^ Ω cm. The system could be expected to provide promising advances in the development of a mild and sustainable fabrication procedure for ITO thin films under mild atmospheric conditions without the use of expensive vacuum production systems or harmful and environmentally undesirable chemicals.

## Introduction

Transparent conductive oxides (TCOs) electrodes on substrates in electronic devices, such as flat panel displays, tablet PCs, mobile phones, and solar cells, are widely manufactured by sputtering followed by etching. This procedure is advantageous for fabricating highly transparent conductive electrodes on substrates with low resistivity and high toughness. However, the sputtering-etching process requires expensive vacuum production systems and the use of harmful and environmentally undesirable chemicals such as oxalic and nitric acids. In contrast, printed electronics (PE) technology^1–6^ has attracted a great deal of attention as an alternative ecofriendly and low-cost process to write transparent electric electrodes on not only glass but also flexible plastic substrates with TCO nanoparticle (NP)-based nanoinks. To date, dip coating^7^, ink-jet printing^8–10^, brush printing^11,12^, and spin coating^13^ have been developed to obtain TCO thin films from NP-based nanoinks. However, the performance of nanoinks as key materials has not been satisfactory from the viewpoint of practical use applicable to fabricate TCO thin films for commercial electronic devices by PE technology. For the preparation of nanoinks, additives such as surfactants and polymers are required to introduce high dispersion stability to the nanoinks. Additives are also applied to provide high adhesion stability of TCO films to substrates. These additives can significantly impair the original low resistivity of the TCO NPs after thin film preparation because the additives covered on the surfaces of the TCO NPs as an insulating layer led to a drastic increase in the interfacial resistance between the TCO NPs^8^. The insulating property of the additives is the most serious problem in obtaining high performance TCO thin films by the ink coating method. A high-temperature heat treatment above 300 °C after casting of TCO nanoink onto a substrate is a simple and effective way to remove the additives; however, such heat treatment is not applicable to the fabrication of flexible devices^14,15^ due to the low heat resistivity of flexible plastic films. From the viewpoint of the above-mentioned current situation, if a TCO film with low resistivity can be manufactured in an atmospheric and ecofriendly system without any additives, then this method will be a promising and innovative process for the preparation of next-generation flexible devices. Recently, a mist-assisted deposition method without the use of additives has attracted much attention as an ecofriendly, atmospheric, and sustainable fabrication process to obtain functional thin films on substrates^16^. In this method, the coating liquid is converted into a mist by ultrasonic irradiation, and the size of the droplets in the mist is tuned on the micrometer order to stabilize them in the mist state in air. This aspect is different from the well-known spray coating method^17^ and NP-chemisorption printing^18,19^. The advantage of the mist-assisted deposition method is that the droplets can be uniformly transported to the surface of a substrate by a carrier gas, and homogeneous films can be produced by rectification of the carrier gas containing misted coating dispersions. Among the deposition methods, mist-assisted chemical vapor deposition using precursor solutions enables us to prepare thin films on substrates via gas phase growth, as the solvent vaporizes immediately before the droplet reaches the substrates. To date, the method has been applied to prepare zinc oxide (ZO) films^20^, solar cells^21–23^, sensors^24–26^, transistors^27^, and SnO_2_-based^28,29^, In_2_O_3_-based^28–32^, and ZnO-based TCO^32–34^ thin films. However, only a few studies about employing the mist-assisted deposition method for functional thin films using NP dispersions have been reported. Watanabe^35^ and Kim^36^ et al. reported the preparation of porous TiO_2_ films from preheated and dried spray mists, respectively. More recently, we demonstrated the preparation of gallium-doped zinc oxide (GZO) on glass substrates as TCO thin films by the GZO NP-mist deposition method^37^. Here, GZO NPs controlled in mean particle size were originally synthesized by a solvothermal method using a mixture of NaOH/NaOMe as bases^37^. The transparency, haze, and resistivity of the GZO NP-mist-deposited thin films on glass substrates were more than 99% at 600 nm, 0.3%, and 5 × 10^−2^ Ω cm after annealing at 300 °C, respectively. Despite extensive investigations, heat treatment at 300 °C under a reducing atmosphere was an essential step to induce low resistivity in the GZO NP-based thin films. The reason is due to the high resistivity of the GZO NP powder compacts (3.0 × 10^2^ Ω cm) used for the deposition. These results strongly prompted us to use TCO NPs with low resistivity, which might emphasize the advantages of the deposition method because a heat-treatment-less process is readily applicable to the preparation of TCO thin films on flexible films under atmospheric conditions. For the TCO NPs, we recently reported that solvothermally prepared single-crystalline cubic-shaped^38–40^ and protrusion-rich indium tin oxide (ITO)^41^ NPs show low resistivity. The electrical resistivity of the powder compacts of the ITO NPs with protrusions under an applied pressure of 20 kN was 1.98 × 10^–2^ Ω cm^41^. Furthermore, compared with the ITO NPs with a cubic shape^38–40^, the ITO NPs with protrusions on their surfaces demonstrated unprecedented high water dispersion stability without adding dispersants such as surfactants. ^1^H nuclear magnetic resonance relaxation measurements revealed that such high dispersion stability in water could be due to the shape-triggered increase in the hydrophilicity of the surfaces of the ITO NPs^41^. This property might be positively applicable to the mist deposition method using water dispersions of ITO NPs. Here, we report the application of solvothermally prepared single-crystalline protrusion-rich ITO NPs with different tin doping amounts to the ITO NP-mist deposition method for the fabrication of ITO thin films on flexible films under atmospheric conditions at room temperature and normal pressure. The NP-mist deposited ITO thin films exhibit low resistivity and high transparency in the visible light region on flexible films, and the resulting films could be applicable for practical use as TCO electrodes.

## Results and discussion

### Synthesis and characterization of ITO NPs for NP-mist deposition

**Figure 1:**
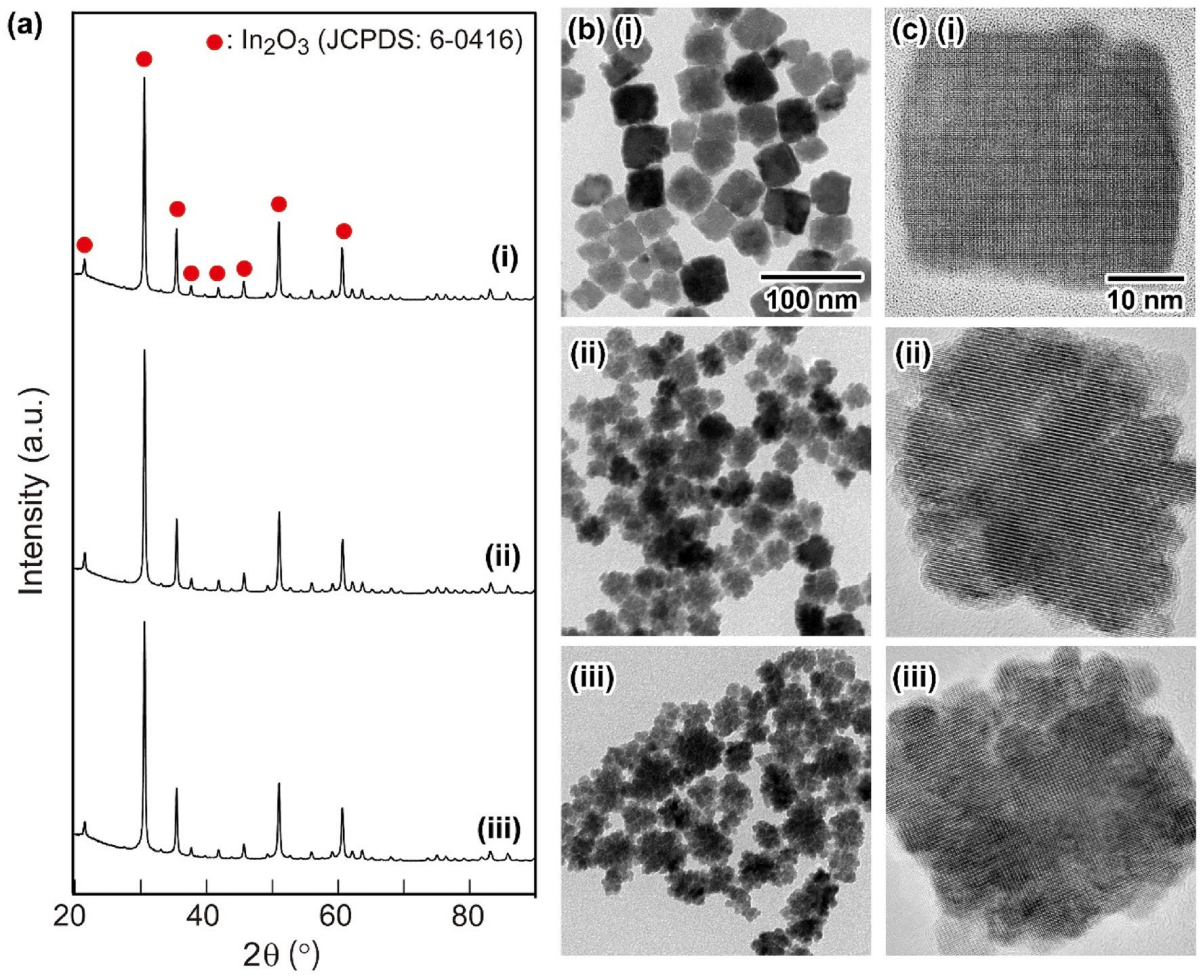
(**a**) XRD patterns of the ITO samples for the NP-mist deposition method: (i) **C1**, (ii) **P1**, and (iii) **P2**. (**b**) TEM images of (i) **C1**, (ii) **P1**, and (iii) **P2**, and (c) HR-TEM images of (i) **C1**, (ii) **P1**, and (iii) **P2**.

**Figure 2:**
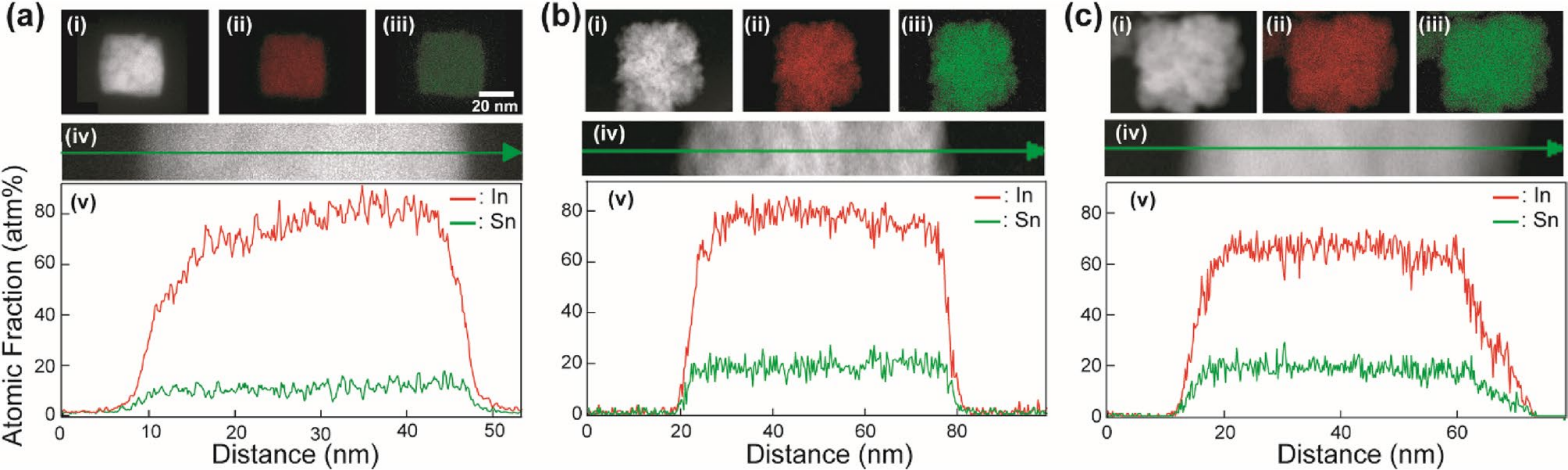
Results of (i) HAADF-STEM analysis of (**a**) **C1**, (**b**) **P1**, and (c) **P2**. EDS mapping images ((ii) indium *K*_α1_; (iii) tin *K*_α1_) of the HAADF-STEM images shown in (**a**), (**b**), and (**c**). (iv) Images showing the positions of the line profiles for determining the distributions of In and Sn atoms in the NPs. (v) Line profiles of In and Sn atoms in the ITO NPs. The maximum intensity of 100 atom% is normalized to the point where the sum of the In and Sn intensities is the highest. The scale bar shown in (**a**) (iii) is common to (i)-(iii).

As the ITO NPs for the NP-mist deposition, 14 mol% Sn-doped ITO NPs with a cubic shape (**C1**), 14 mol% Sn-doped ITO NPs with protrusions on their surfaces (**P1**), and 25 mol% Sn-doped ITO NPs with protrusions (**P2**) were used in the present study. **C1** and **P1** were synthesized based on our previous report^41^. After optimizing the reaction conditions, **P2** was prepared by a procedure similar to that for the preparation of **P1**. The details of the preparation of **C1** and **P2** are summarized in the following Methods section (for **P1**, *see* ref. 41). Figure 1a (i), (ii), and (iii) shows the X-ray diffraction (XRD) patterns of before annealed **C1**, **P1**, and **P2**, respectively. All sharp diffraction peaks in Fig. 1a (i)-(iii) can be assigned to the formation of an In_2_O_3_ crystal structure (JCPDS No. 6-0416) with high crystallinity, indicating that the obtained particles **C1**, **P1**, and **P2** were in a single phase. The molar ratios of [Sn^4+^]/[In^3+^] in **C1**, **P1**, and **P2** were calculated from the results of inductively coupled plasma-atomic emission spectrometry (ICP-AES) measurements. From the results, the doping amounts of [Sn^4+^] in **C1**, **P1**, and **P2** could be assigned as 14.4 mol%, 14.2 mol%, and 24.8 mol%, respectively, based on [In^3+^]. These doping molar ratios of [Sn^4+^] were close to the initial mixing molar ratios of [Sn^4+^]/[In^3+^]: 0.122, 0.122, and 0.247 for **C1**, **P1**, and **P2**, respectively. The doping amounts of Sn ions in the ITO crystals well reflected the initial [Sn^4+^]/[In^3+^] ratios. Transmission electron microscopy (TEM) and high-resolution TEM (HR-TEM) images of the as-prepared particles **C1**, **P1**, and **P2** are shown in Fig. 1b. **C1** had a cubic shape with smooth edge surfaces (Fig. 1b (i)). The mean particle size and distribution of **C1** were 39 ± 12 nm. In contrast, **P1** and **P2** exhibited a protrusion shape (Fig. 1b (ii) and (iii)). The mean particle size and distribution shown in Fig. 1b (ii) and (iii) were 38 ± 10 nm and 43 ± 10 nm, respectively, determined by counting 100 particles in the TEM images. From the HR-TEM images exhibited in Fig. 1c (i)-(iii), clear and uniform fringes corresponding to basically single-crystalline crystal structures were seen for all the NPs, especially **P1** and **P2**, which have protrusion-rich rough surfaces. The crystallite sizes of **C1**, **P1**, and **P2** were calculated as 35 nm, 33 nm, and 34 nm, respectively, by Scherrer's equation. The sizes were in good agreement with the mean particle diameters determined by the TEM observations. The results also suggested that **C1**, **P1**, and **P2** had basically single-crystalline structures. Figure 2 presents the characterization results for the internal structures and surface states of **C1**, **P1**, and **P2** obtained using high-angle annular dark field scanning TEM (HAADF-STEM) equipped with an energy dispersive X-ray spectroscopy (EDS) system. Figure 2a, b, and c shows the HAADF-STEM analysis results of **C1**, **P1**, and **P2**, respectively. The EDS mapping images of In and Sn atoms seen in (ii) and (iii), respectively, for the ITO NPs revealed that Sn ions were uniformly distributed in the NPs. The line profiles of the distribution of In and Sn atoms in the NPs are summarized in (v). These results supported that Sn^4+^ was successfully uniformly doped into the In_2_O_3_ crystal structure. The resistivities of the powder compacts of as-prepared **C1**, **P1**, and **P2** were 1.1 × 10^1^ Ω cm, 5.8 × 10^−1^ Ω cm, and 8.6 × 10^−1^ Ω cm under application of a pressure of 20 kN, respectively. After heat treatment at 300 °C for 0.5 h under a 4% H_2_ atmosphere, the resistivities of the powder compacts of **C1**, **P1**, and **P2** decreased to 3.5 × 10^−2^ Ω cm, 2.0 × 10^−2^ Ω cm, and 7.9 × 10^−2^ Ω cm, respectively.

### The ITO NP-mist deposition method

**Figure 3:**
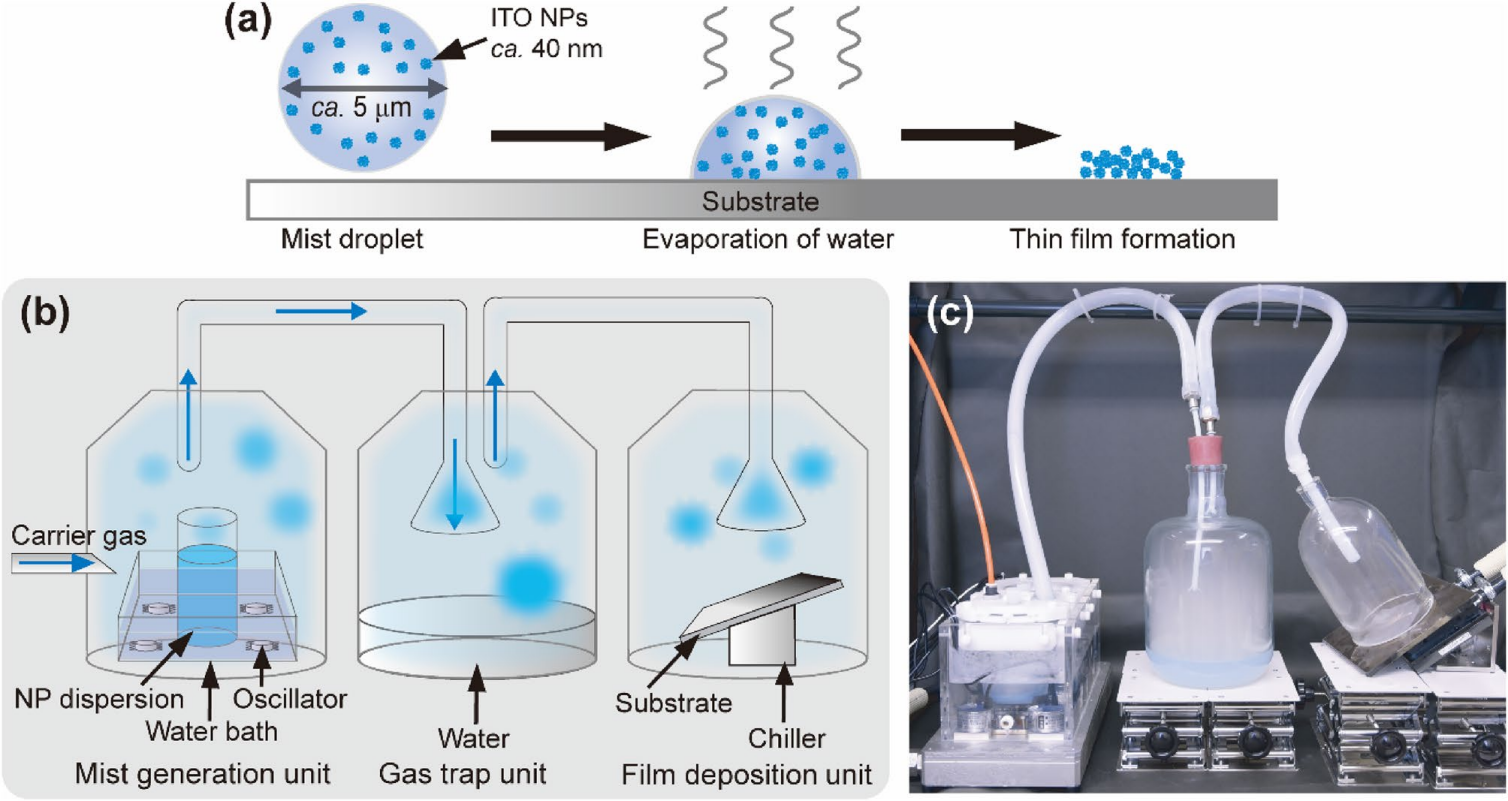
(**a**) Schematic image of the ITO NP-mist deposition mechanism on substrates. (**b**) Illustration and (**c**) photo of the setup for the ITO NP-mist deposition method developed in the present study.

As mentioned above, the NP-mist deposition method enables us to obtain NP-based homogeneous thin films by controlling the deposition speed of the NPs in the droplets of the mist. The deposition speed is readily tuned by the flow rate of the carrier gas and the applied voltage of the ultrasonic irradiator to generate mists. By controlling the frequency of the ultrasonic oscillator, a variety of solvents can be converted into mists to deposit the NPs in thin film states. Figure 3a exhibits a schematic image of the deposition mechanism on substrates. Figure 3b and 3c shows an illustration and a photo of the setup for the ITO NP-mist deposition method. The deposition system consists of a mist generation unit, a gas trap, and a film deposition unit (Fig. 3b). The mist generation unit is a thermostatic water bath equipped with four ultrasonic oscillators in the bottom of the bath. The NP-mist is generated by immersing a plastic bottle with the ITO NP dispersion (ca. 100 mL) into the mist generation unit. The ITO NP-mist is transported to the gas trap by introducing a carrier gas with a controlled flow rate into the bottle. The large droplets and dust in the mist, preventing homogeneous film preparation, are removed in the gas trap unit. As a result, ITO NP-mist controlled to a few microns in size with a narrow size distribution is supplied to the film deposition unit. According to Kelvin’s equation^42^, the vapor pressure of the small water droplets becomes high, and water in the droplets is readily volatized immediately after contact with the substrates, depositing the ITO NPs on the substrates to form uniform and densely packed ITO thin films with high adhesion stability (*see* Fig. 3a). The deposition speed is also tuned by controlling the temperature of the substrates in the deposition unit. Cooling or heating the substrates induces an increase or a decrease in the deposition rate, respectively. The remaining ITO mist not used for deposition can be collected by an air filter and recycled with high efficiency. Furthermore, by designing the shape of the deposition nozzle in the deposition unit, ITO thin films can be uniformly fabricated on not only flat but also curved substrates.

### Effect of the deposition method on the resistivity of the ITO thin films

**Figure 4:**
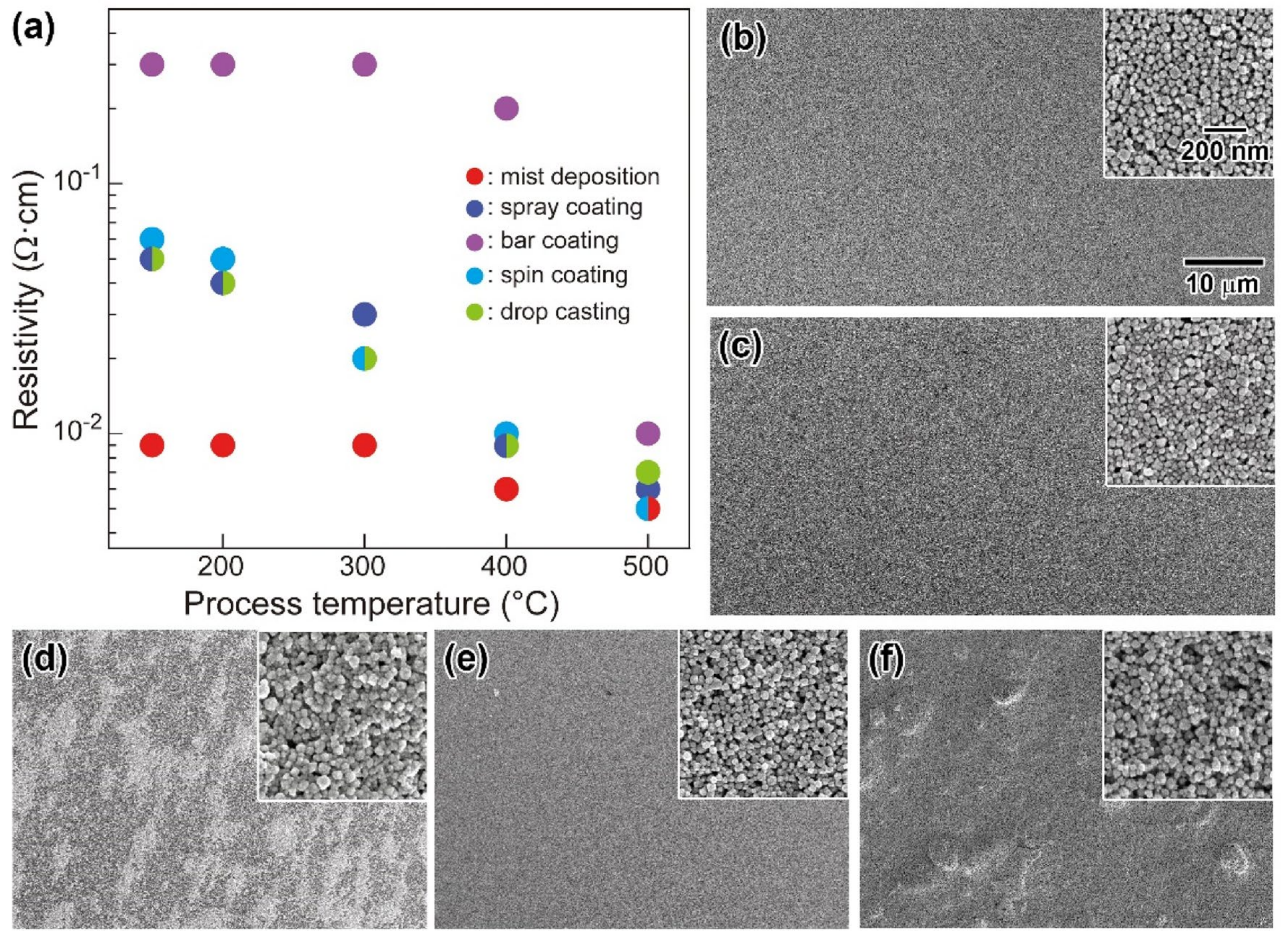
(**a**) Resistivity of the **P1** thin films on glass substrates obtained by the different preparation methods. The resistivity was measured after annealing at various process temperatures in Ar-H_2_ (4% H_2_) for 1 h. SEM images of the surfaces of the **P1** thin films on glass substrates prepared by the (**b**) mist deposition (**c**) spray coating, (**d**) bar coating, (**e**) spin coating, and (**f**) drop casting methods. These images were taken after annealing at 150 °C. The insets in (**b**)–(**f**) are close-up images of the surfaces. The scale bar shown in (**b**) is common to all images.

To investigate the effect of the coating method on the resulting resistivity of the ITO thin films, **P1** NP-based thin films were deposited on glass substrates by NP-mist deposition, bar coating, spray coating, spin coating, and drop casting. Unless otherwise noted, a water dispersion of **P1** (5 wt% in water) was applied. Figure 4a shows the resistivities of **P1** thin films prepared by the different preparation methods after heat treatment at 150, 200, 300, 400, and 500 °C for 1 h under a H_2_-Ar (4% H_2_) atmosphere. Notably, the resistivity of the thin film obtained by the mist deposition method was the lowest, reaching 9.0 × 10^−3^ Ω cm after heat treatment at 150 °C. In contrast, the bar-coated **P1** film showed the highest resistivity at 3.0 × 10^−1^ Ω cm. The resistivities of the corresponding films prepared by the spray coating, spin coating, and drop casting methods were 4.0 × 10^−2^ Ω cm, 5.0 × 10^−2^ Ω cm, and 4.0 × 10^−2^ Ω cm, respectively, at the same process temperature. The low resistivity at a low process temperature, only observed for the NP-mist deposition method, is quite a strong advantage of applying the NP-mist deposition method to fabricate **P1** films on flexible films with low heat-resistance properties. The resistivity of the thin films decreased with increasing heat treatment temperature for all cases. After annealing at 500 °C, the resistivities of all the thin films reached less than 1 × 10^−2^ Ω cm, and the lowest value of 5.0 × 10^−3^ Ω cm was observed for the NP-mist-deposited thin film. Figure 4b, c, d, e, and f presents scanning electron microscopy (SEM) images of the surfaces of the **P1** thin films prepared by the mist deposition, spray coating, bar coating, spin coating, and drop casting methods, respectively. These images were taken after annealing at 150 °C. From the SEM image, the **P1** films with flat and smooth surfaces were prepared by the mist deposition, spray coating, and spin coating methods. In contrast, the surface of the **P1** thin film fabricated by bar coating and drop casting methods formed rough states with aggregated structures over the entire surface. Atomic force microscopic (AFM) observations of the corresponding **P1** thin films revealed that the roughness (*R*_a_) of the surfaces of the **P1** thin films prepared by NP-mist deposition, bar coating, spray coating, spin coating, and drop casting methods could be assigned as *R*_a_ = 7.6 nm, 7.8 nm, 10.0 nm, 15.8 nm, and 12.6 nm, respectively (see, supplementary information (SI), Figure S1). Tang et al. reported that the relationship between the *R*_a_ and the resistivity of the ITO thin films, with the thickness of 150 nm, prepared by a sputtering method^43^, and the resistivity was decreased by the decreasing *R*_a_. In the present report, similar behavior was observed by the comparison of *R*_a_s of the **P1** films with flat and smooth surfaces prepared by the mist deposition (*R*_a_ = 7.6 nm), spray coating (*R*_a_ = 10.0 nm), and spin coating (*R*_a_ = 15.8 nm) methods. On the other hand, the highest resistivity (3.0 × 10^−1^ Ω cm) of the drop-cast thin film after annealing at 150 °C could be due to the existence of ethylene glycol used for the solvent for the coating in the film. As above mentioned, in the case of the NP-mist deposition method, the evaporation rate of the liquids in the droplets can be readily adjusted by controlling the substrate temperature and the carrier gas flow rate, which suppresses the agglomeration of NPs in the droplets of the mist and on the substrates to form uniform thin films. The insets of Fig. 4b–f are the close-up images of the corresponding surfaces of the **P1**-based thin films. The densely-packed uniform surface is recognized from the images shown in Fig. 4b. This result indicates that the NP-mist deposition method has great advantages in the deposition of ITO NP aqueous dispersions on substrates for fabricating high-performance ITO thin films with densely-packed, flat, and smooth surfaces at low processing temperatures applicable to flexible films.

### Characterization of NP-mist-deposited ITO thin films prepared using C1 and P1

**Figure 5:**
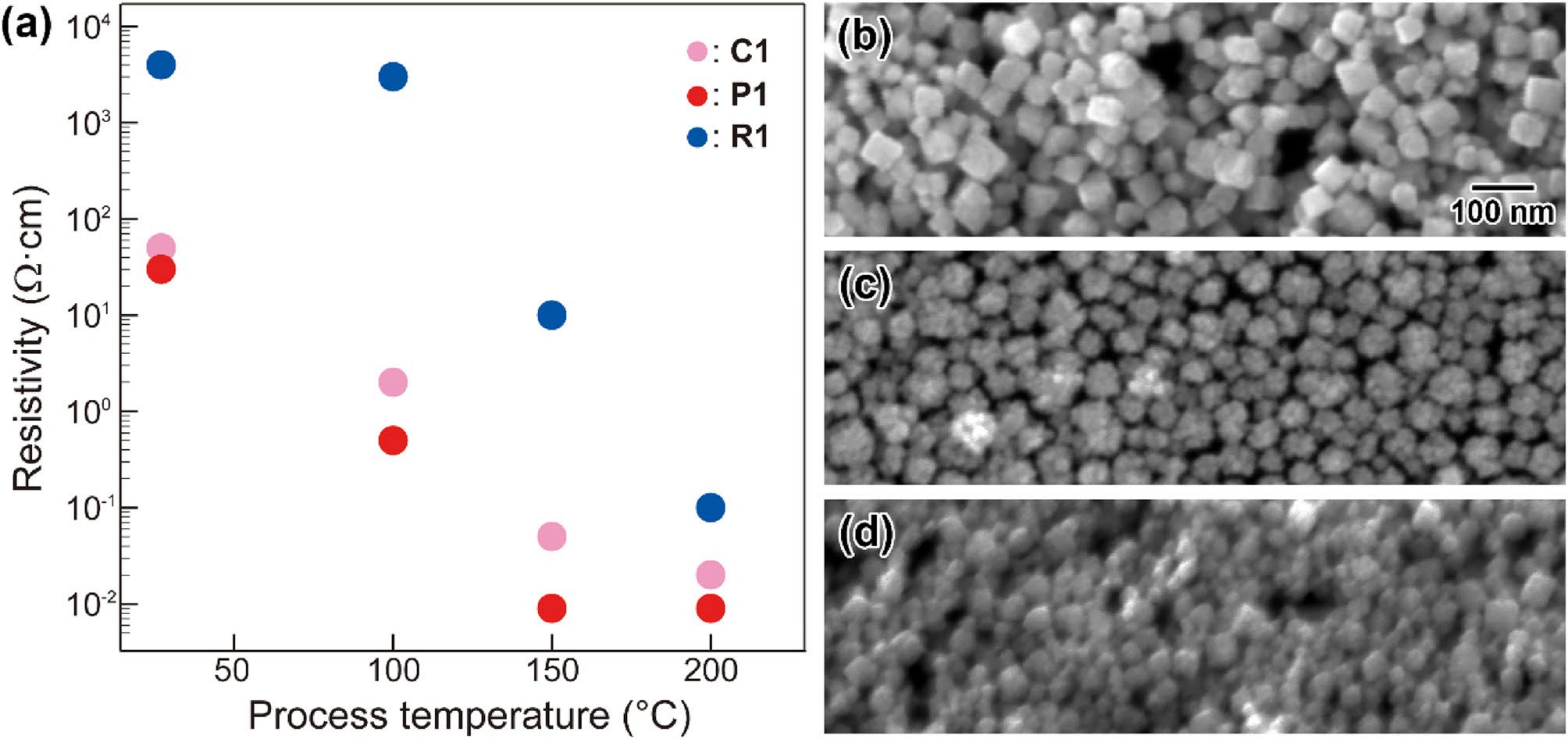
(**a**) Resistivity of the **C1**-, **P1**-, and **R1**-based thin films on glass substrates obtained by NP-mist deposition in a low process temperature range from room temperature to 200 °C. SEM images of the surfaces of the (**b**) **C1**-, (**c**) **P1**-, and (**d**) **R1**-based ITO thin films prepared by the NP-mist deposition method after annealed at 150 °C. The scale bar shown in (**b**) is common to (**c**) and (**d**).

**Figure 6:**
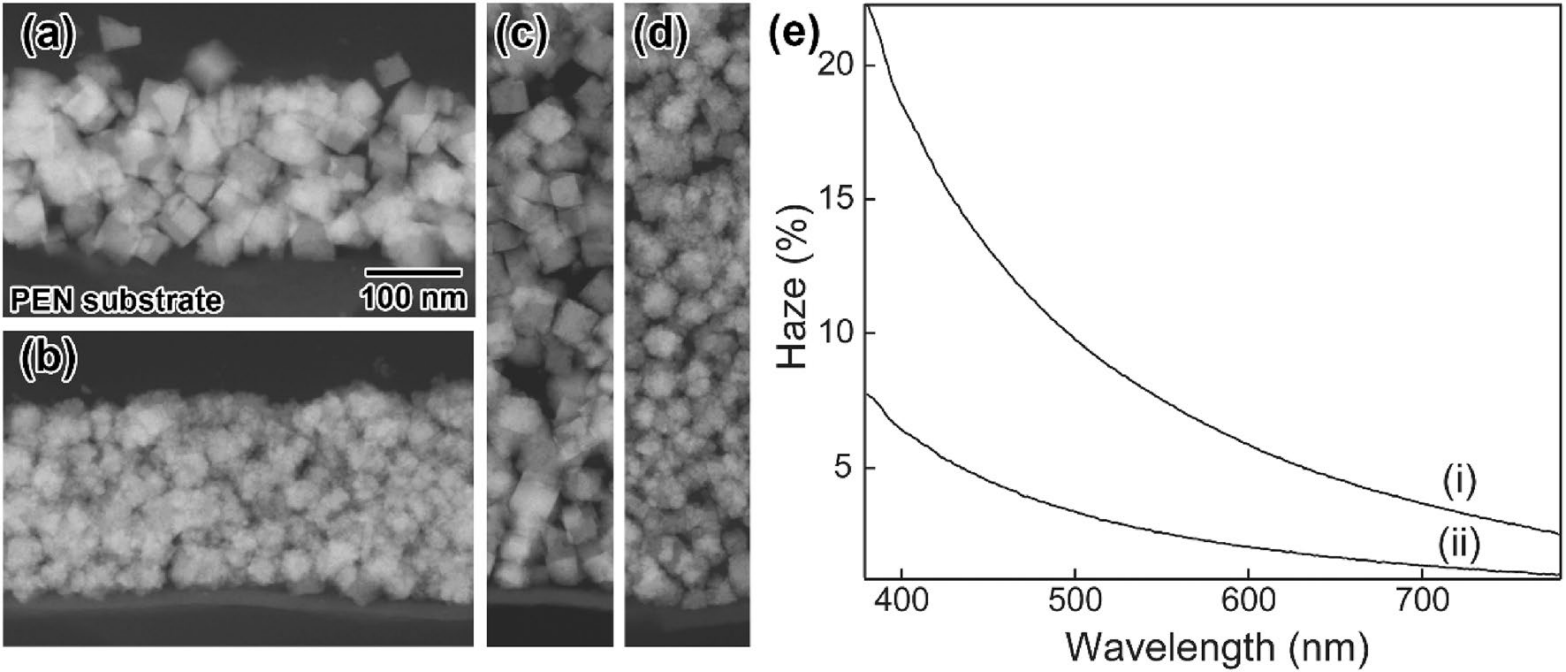
Cross-sectional scanning TEM images of ITO thin films prepared by the NP-mist deposition method using (**a**) **C1** and (**b**) **P1** on PEN substrates. The thickness was controlled to *ca.* 200 nm. Corresponding images of the thick films of (**c**) **C1** and (**d**) **P1** with thicknesses of *ca*. 500 nm. (**e**) Results of haze measurements of the (i) **C1-** and (ii) **P1**-based thin films on PEN substrates. The scale bar shown in (**a**) is common to (**b**)-(**d**).

Next, ITO thin films were prepared by the NP-mist deposition method using **C1**- and **P1**-based water dispersions, and the resistivities and surface states of the resulting ITO thin films on glass substrates were characterized. As a reference sample, a commercially available water dispersion (15 wt%) of ITO NPs was purchased (containing 5 wt% polymer dispersants) and applied for deposition. The ITO NPs in this dispersion were abbreviated as **R1**. Figure 5a summarizes the resistivities of the **C1**-, **P1**-, and **R1**-based thin films prepared by NP-mist deposition in a low process temperature range from room temperature to 200 °C. The resistivities of the **R1**-based thin films were more than *ca*. 100 times those of the corresponding **C1**- and **P1**-based thin films at process temperatures from room temperature to 150 °C. Figure 5b, c, and d presents SEM images of the surfaces of the **C1**-, **P1**-, and **R1**-based thin films, respectively. The corresponding AFM images are shown in Figure S2. The SEM and AFM images were taken after annealing at 150 °C. A relatively densely packed uniform and flat surface state was seen for the **R1**-based film (Fig. 5d and Figure S2b). A plausible reason why the **R1**-based ITO thin film had high resistivity despite the packing state might be the existence of the organic dispersants in the dispersion, and the dispersants might remain in the film, inducing an increase in the interparticle resistivity between **R1**. To further decrease the resistivity of **R1**-based thin films, heat treatment at 200 °C to remove organic components in the films was an essential step. These results suggested that the **R1** dispersion was not applicable to preparing ITO thin films on flexible plastic films. In contrast, the resistivities of the **C1**- and **P1**-based thin films after annealing at 150 °C were 8.0 × 10^−2^ Ω cm and 9.0 × 10^−3^ Ω cm, respectively. The low resistivity at a low process temperature enabled us to produce ITO thin films on plastic films, as mentioned below. Both water dispersions of **C1** and **P1** contain no organic dispersants, and the fundamental difference between **C1** and **P1** is the shape-induced dispersion stability in water over a long period^41^. The SEM images in Fig. 5b revealed that the surface state of the **C1**-based thin film was totally different from that of the corresponding **P1**-based thin film. Rough and porous structures were clearly observed in Fig. 5b. In contrast, a uniform and flat surface state was seen for the **P1**-based ITO thin film (Fig. 5c). The lower resistivity of the **P1**-based ITO thin film than that of the corresponding **C1**-based film can derive from the densely packed state of the **P1**-based thin film. Figure 6a and b presents cross-section TEM images of **C1**- and **P1**-based ITO thin films on polyethylene naphthalate (PEN) films (TEONEX, Q51-188, TOYOBO Co., Ltd., *t* = 188 μm) with thicknesses of *ca*. 200 nm, respectively. Figure 6c and d shows the corresponding cross-section TEM images of **C1**- and **P1**-based ITO thick films with thicknesses of *ca*. 500 nm, respectively. These images well reflected the corresponding SEM images shown in Fig. 5b and c. Loosely deposited porous structures with voids and irregularities, seen in the SEM image (Fig. 5b), were also observed inside the **C1**-based film (Fig. 6a and c). Densely deposited **P1**-based structures were not only formed on the surface of the thin film but also fabricated throughout the interior of the thin film (compare Figs. 5c and 6b–d). The difference in the deposition states between the **C1**- and **P1**-based ITO thin films is thought to be due to the difference in the dispersion states of **C1** and **P1** in water droplets of the mists. For direct observation of the dispersion states of **C1** and **P1** in water droplets, small angle X-ray scattering (SAXS) measurements were carried out. The characterization results shown in Figure S3 revealed that the average mean particle size and distribution of the particles in the **P1**-based droplets were 41 ± 13 nm. These values were quite consistent with the size determined by TEM observation (38 ± 10 nm). This result clearly suggested that **P1** existed as primary particles in the droplets (a similar result was observed for **P2**: SAXS: 45 ± 16 nm; TEM: 43 ± 10 nm). In contrast, two types of particles were seen in the droplets prepared from a water dispersion of **C1**. The size and distribution of the particles were 54 ± 10 nm and 159 ± 30 nm. The smaller type corresponded to the **C1** particles dispersed as primary particles, and the other type corresponded to the coalesced secondary particles of **C1** in the droplets (**C1** from TEM: 39 ± 12 nm). The ratio of the primary and secondary particles in the **C1**-based droplets could be assigned as 1/4. As mentioned above, **P1** shows high dispersion stability in water for a long period while maintaining a primary particle state in water. From the SAXS measurements, this property was also maintained in the water droplets. The resulting continuous deposition of **P1** in a primary particle state might enable us to obtain thin films with densely packed interior structures on the substrates. In contrast, the low water dispersion stability of **C1** probably induced the formation of aggregated secondary particles in the water droplets and prevented the formation of a densely packed thin film on the substrates.

Figure 6e(i) and (ii) shows the results of haze measurements of the **C1**- and **P1**-based thin films on PEN substrates, respectively. The haze of the **P1**-based thin film is much lower than that of the **C1**-based thin film in the visible light region. This result indicated that the **P1**-based thin film had a lower light scattering property than the **C1**-based thin film. The low haze property is due to the uniform and flat surface state and densely deposited interior structures of the **P1**-based thin film, which reduce the light scattering. These results strongly suggested that the high dispersion stability of **P1** in water played an important role in fabricating high-performance ITO thin films with low resistivity and low haze property by the NP-mist deposition method.

### ITO NP-mist deposition on flexible films

**Figure 7:**
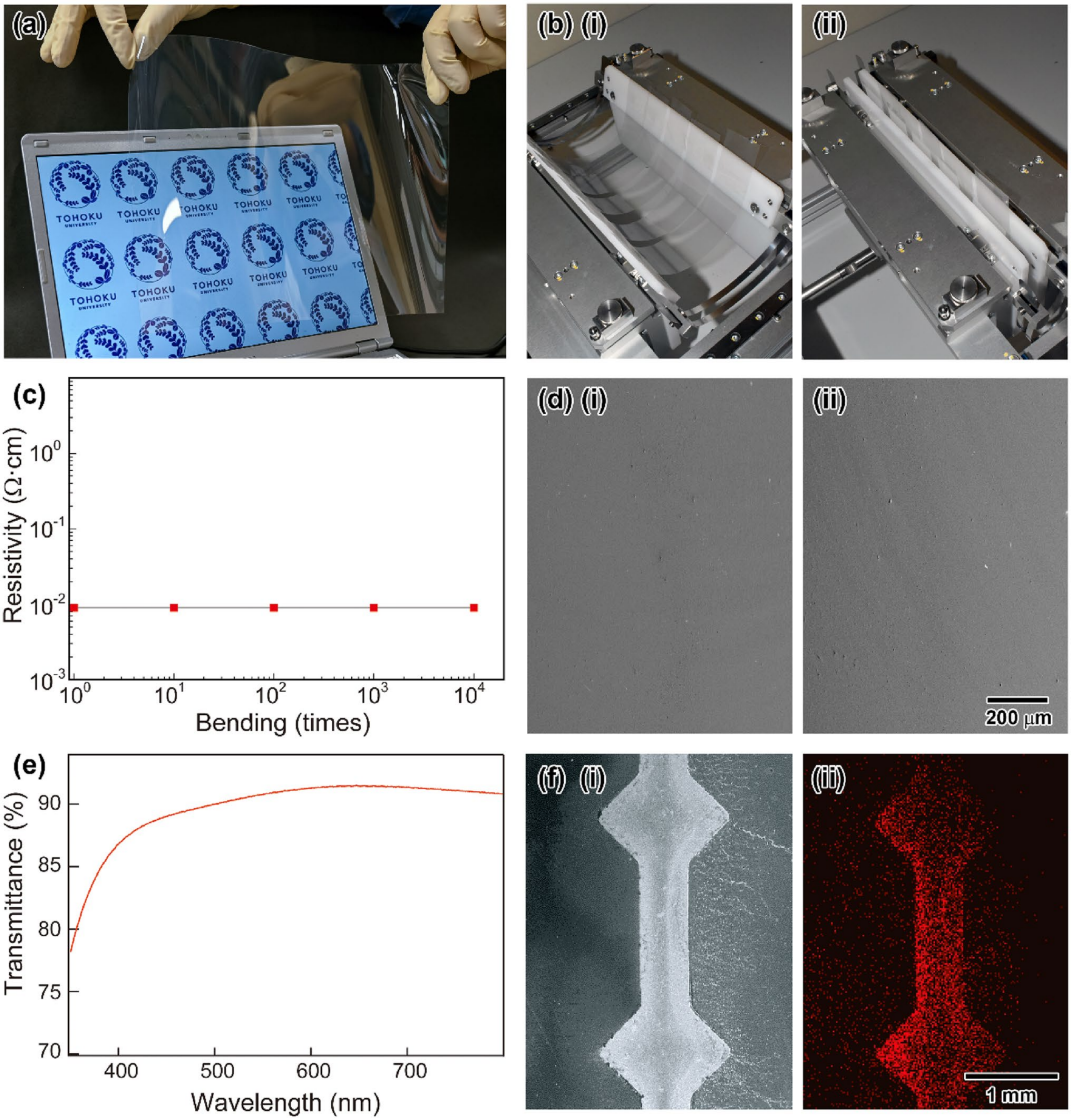
(**a**) Appearance of an ITO thin film (ca. 300 nm) on a PEN substrate (Q51-50, TOYOBO Co., Ltd., *t* = 50 μm) deposited by the NP-mist deposition method using **P1**. (**b**) Photos of the ITO thin films on PEN substrates (i) before and (ii) after bending. (**c**) Change in the resistivity with bending. (**d**) SEM images of the **P1**-based ITO thin films (i) before and (ii) after bending 10,000 times. The scale bar shown in (ii) is common to (i). (**e**) Transmittance spectra of the **P1**-based ITO thin film on a PEN substrate prepared by the NP-mist deposition method (background: the corresponding PEN film). (**f**) (i) SEM image and (ii) corresponding EDS mapping image of In ions for the **P1**-based thin film on a PEN substrate. The scale bar shown in (ii) is common to (i).

The ITO NP-mist deposition method was applied to fabricate ITO NP-based flexible thin films. The experimental details are summarized in the Methods section. Figure 7a shows a photo of an ITO flexible thin film on a PEN substrate prepared by the NP-mist deposition method using **P1**. The thickness of the ITO thin film was adjusted to 300 nm by controlling the deposition period. The photo was taken after annealing at 150 °C for 1 h under a N_2_ atmosphere. The resistivity of the resulting highly transparent and flexible film was 9.0 × 10^−3^ Ω cm, and similar resistivities were observed on glass substrates. To investigate the mechanical durability of the thin films, bending tests with an angle of 5° were carried out. Figure 7b (i) and (ii) shows photos before and after bending, respectively. After 10,000 bending cycles under atmospheric conditions, no increase in the resistivity was observed for the films (Fig. 7c). Figure 7d (i) and (ii) exhibits SEM images of the **P1**-based thin films before and after bending 10,000 times. No cracks, detachment, or voids were seen in the images. The results showed that **P1** had high adhesion stability to the flexible films. Figure 7e exhibits the transmittance spectra of the **P1**-based thin film on a PEN substrate. The transmittance of the **P1**-based thin film was more than 85% in the region from 400 to 800 nm, and a highly transparent ITO thin film could be obtained by the NP-mist deposition method under the mild and sustainable fabrication procedure. Further, by pre-hydrophilic treatment of the flexible films, ITO patterned films were fabricated by the present method. The pattern for selective deposition was realized as follows: Initially, NOVAC 1720 (3 M) was precoated on a polyethylene terephthalate (PET) substrate (COSMOSHINE, A4160, TOYOBO Co., Ltd., *t* = 188 μm), a PEN film (TEONEX, Q51-50, TOYOBO Co., Ltd., *t* = 50 μm), or a PEN film (TEONEX, Q51-188, TOYOBO Co., Ltd., *t* = 188 μm) by the mist deposition system to prepare flexible films with hydrophobic surfaces. The films were dried at 100 °C for 30 min. Then, the films were masked by a patterned SUS430 thin substrate (*t* = 100 μm). The resulting masked flexible PET and PEN films were treated by a UV-ozone cleaner for 5 min to obtain the patterned hydrophilic substrates. Figure 7f (i) and (ii) exhibits an SEM image and the corresponding EDS mapping image of In ions for the **P1**-based thin film on a PEN substrate, respectively. The water droplets in the mist spontaneously deposited only on the hydrophilized surface of the flexible films to form a uniform pattern. The selective and spontaneous process will become a powerful technique for fabricating ITO patterned films under ecofriendly atmospheric roll-to-roll PE conditions.

### Effect of Sn doping amount on the properties of ITO NP-based thin films

**Figure 8:**
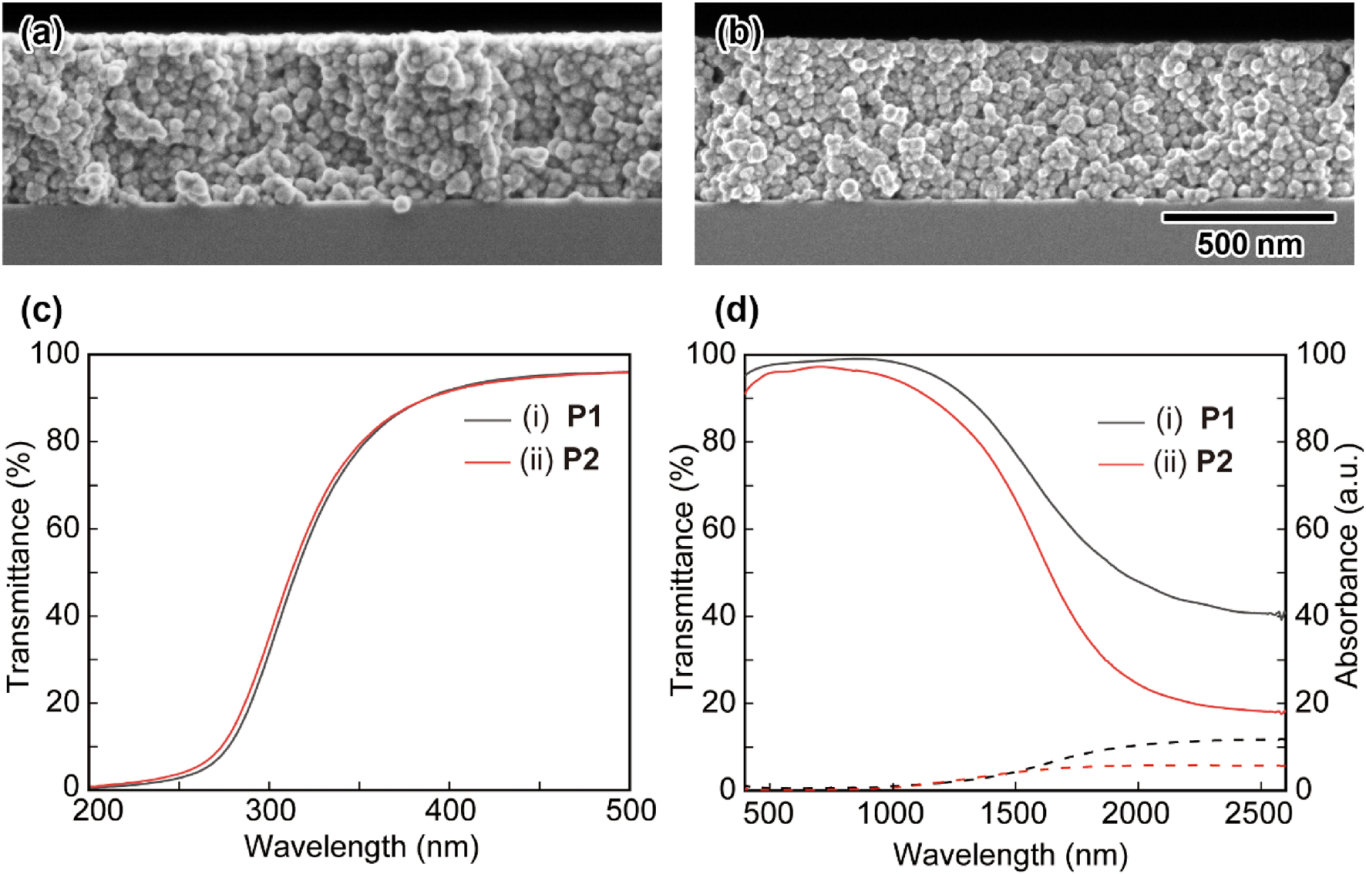
Cross-section SEM images of the (**a**) **P1**- and (**b**) **P2**-based ITO thin films on silicon substrates obtained by the NP-mist deposition method. The scale bar shown in (**b**) is common to (**a**). (**c**) Transmittance and (**d**) UV-NIR spectra of the (i) **P1**- and (ii) **P2**-based ITO thin films on glass substrates. Solid lines: transmittance; dashed lines: absorbance.

By tuning the reaction conditions, we successfully prepared **P2** with a Sn doping amount of 25 mol% based on [In^3+^]. Figure 8(a) and (b) shows cross-section SEM images of the ITO thin films on silicon wafers deposited by using **P1** and **P2**. Both the **P1**- and **P2**-based thin films had densely packed states with no voids, indicating that the change in the doping amount of Sn ions in the In_2_O_3_ crystal structure had little effect on the film structure. Figure 8 (c) exhibits transmittance spectra of the (i) **P1**- and (ii) **P2**-based thin films in the short wavelength region after annealing at 150 °C in Ar-H_2_ (4% H_2_) for 1 h, where the absorption edge shifted to a shorter wavelength with increasing Sn doping amount. This result showed that the band gap differed depending on the Sn doping amount, indicating that Sn was effectively doped into the ITO NPs and that the band gap could be controlled by adjusting the doping amount of Sn ions. The calculated band gaps^29^ of the **P1**- and **P2**-based thin films were 3.85 eV and 3.95 eV, respectively. UV-NIR spectra of the **P1**- and **P2**-based thin films after heat treatment at 150 °C in Ar-H_2_ (4% H_2_) for 1 h are summarized in Fig. 8d. In the wavelength region above 1500 nm, the reflectance of the **P2**-based thin film was higher than that of the **P1**-based thin film. Since the reflection in this region is due to conduction carriers in the ITO crystal, this result suggested that the carrier concentration increased with increasing Sn doping amount. Hall mobility measurement is an efficient method to ITO thin films^44,45^. However, the resistivities of the **P1**- and **P2**-based thin films after annealing at 150 °C in Ar-H_2_ (4% H_2_) for 1 h were 9.0 × 10^−3^ Ω cm (270 Ω/sq.) and 8.5 × 10^−3^ Ω cm (255 Ω/sq.), respectively. These values are still too high for Hall mobility measurement. Further characterization of the effect of Sn doping amount on the electro-optical properties of the ITO thin films are now in progress. On the other hand, the resulting **P1**- and **P2**-based thin films could be kept for more than two weeks under a N_2_ atmosphere at room temperature without changes in the resistivity. We further calculated values of figure of merit (FOM)^46^ to compare the performance of the **P1**- and **P2**-based thin films with previous studies of TCO materials. For the calculation, average transparency from 400 to 800 nm of 96% and 95% for the **P1**- and **P2**-based thin films, respectively, was used. As the resulting values of FOM for the **P1**- and **P2**-based thin films were 38 and 36, respectively. These values were comparative to the ITO thin films obtained by a sputtering method (e. g. FOM = 40, sheet resistivity: 100 Ω/sq., transparency: 90%)^47^. These results suggested that high-performance ITO thin films were successfully fabricated by the NP-mist deposition method under mild and ecofriendly conditions by using protrusion-rich ITO NPs with high water stability.

## Methods

### Reagents

Unless otherwise noted, all reagents were used as received. Water was doubly distilled, deionized, and filtered prior to use. Indium(III) chloride anhydrous (InCl_3_, Kishida Chemical Co., Ltd., 98%, Cat. No. 020-41392), tin(IV) chloride anhydrous (SnCl_4_, FUJIFILM Wako Pure Chemical Corp., 99.99%, Cat. No. 207-01552) and methanol (MeOH, FUJIFILM Wako Pure Chemical Co., super dehydrated, Cat. No. 131-16777) were used for the ITO NP synthesis. A 1.6 M solution of tetramethylammonium hydroxide (TMAH) in MeOH was prepared by concentrating a 10% MeOH solution of TMAH (Tokyo Chemical Industry Co., Ltd., Cat. No. T0676). A commercially available water dispersion of ITO NPs was purchased from CIK NanoTek Corporation as a reference sample. The ITO NPs in this dispersion were abbreviated as **R1**. The components of the dispersion consisted of **R1** (15 wt%), polymer surfactants (5 wt%), and water (80 wt%).

### Synthesis of ITO NPs for NP-mist deposition

Synthesis of **C1**: Initially, 19.9 g (0.090 mol) of anhydrous InCl_3_ and 1.13 mL (2.6 g) of SnCl_4_ (0.010 mol) were dissolved in anhydrous methanol, and the total volume of the solution was adjusted to 500 mL by the further addition of anhydrous methanol. The resulting concentrations of InCl_3_ and SnCl_4_ in the methanolic solution were 0.180 M and 0.020 M, respectively. This solution was then added to 500 mL of a 1.6 M methanol solution of TMAH in a 2 L Teflon-made vessel and stirred (600 rpm) at room temperature for 10 min. The resulting vessel containing the reaction mixture was put into a high-pressure reactor (Berghof BR-2000, volume: 2 L) and then aged at 190 °C for 24 h without stirring. After cooling to room temperature, the solid particles and the liquid phase thus obtained in the Teflon-made vessel were separated by centrifugation, and the sediments were washed twice with ethanol and twice with ion-exchanged water by dispersal and centrifugation. The blue-colored solid particles were dried at 50 °C in air to obtain **C1**^41^.

Preparation of **P2**: A total of 0.796 g (3.60 × 10^−3^ mol) of anhydrous InCl_3_ and 104 μL of SnCl_4_ (8.88 × 10^−4^ mol) were dissolved in 10 mL of MeOH, and the concentrations of InCl_3_ and SnCl_4_ were adjusted to 0.36 M and 0.089 M, respectively. This solution was then added to 10 mL of a 1.6 M MeOH solution of TMAH and stirred at room temperature for 10 min. The mixture was transferred into a Teflon-lined autoclave (Parr 4749, volume: 23 mL) and then heated at 190 °C for 24 h without stirring. After rapid cooling to room temperature by immersing the autoclave in running water, the resulting blue-colored solid particles and the liquid phase in the autoclave were separated by centrifugation, the sediments were washed twice with ethanol and twice with ion-exchanged water by dispersal and centrifugation, and the resulting solid particles were dried at 50 °C in air to obtain **P2**.

### Preparation of water dispersions of ITO NPs for NP-mist deposition

Water dispersions of **C1**, **P1**, and **P2** for the ITO NP-based mist deposition method for the preparation of ITO thin films were prepared as follows: The powder of **C1**, **P1**, or **P2** (5.0 g) was added to ion-exchanged water (*ca*. 25 mL), and the total volume was adjusted to 100 mL by further addition of water at room temperature. The ITO NPs in the resulting mixtures were well dispersed by a homogenizer (115 W, Amp.: 20 μm, VCX-750, Sonics & Materials, Inc.) for 30 min at room temperature. The concentration of the ITO NPs in the dispersions became 5 wt%. The dispersions of **P1** and **P2** could be kept for more than one month at room temperature without any precipitation of the NPs and could be applied for NP-mist deposition without pretreatment prior to use. For **C1**, the dispersion was unstable in ion-exchanged water, and **C1** settled on the bottom of the vessel after standing for 1 day. Thus, the dispersion of **C1** was dispersed by a homogenizer for 60 min immediately before use in the deposition method.

### Fabrication of ITO NP-based thin films on glass substrates by the NP-mist deposition method

ITO thin films on glass substrates (CORNING, EAGLE XG Slim, thickness: 0.7 mm, alkaline earth boroaluminosilicate) were prepared by the NP-mist deposition system as follows. Initially, all the glass substrates were washed in turn with isopropanol and ion-exchanged water under ultrasonic irradiation. After drying in an oven, the substrates were hydrophilized by treatment with a UV-ozone cleaner (OCA-150L-D, EYE GRAPHICS Co., Ltd.) for 10 min. Then, ITO NP deposition on the substrate was carried out by the custom-made system shown in Fig. 3. The mist generation unit consisted of four ultrasonic oscillator units purchased from SEOKO GIKEN Inc. (1.6 MHz). The ITO NP-dispersion mist formed in the mist generation unit was applied to a glass substrate by a N_2_ carrier gas flow to obtain ITO NP-based thin films on the glass substrate. The flow rate was controlled in a range from 10 to 20 L min^-1^. The resulting thin films were heat treated in air for 1 h at 150 °C and in H_2_-Ar (4% H_2_) for 1 h at 200, 300, 400, or 500 °C. The thickness of the ITO NP-based thin films was adjusted to 300 nm by controlling the deposition period.

### Preparation of ITO NP-based thin films on flexible films by the NP-mist deposition method

ITO NP-based thin films on flexible films were obtained by the following procedure. As the flexible film, a PET substrate (COSMOSHINE, A4160, TOYOBO Co., Ltd., *t* = 188 μm), a PEN film (TEONEX, Q51-188, TOYOBO Co., Ltd., *t* = 188 μm), or a PEN film (TEONEX, Q51-50, TOYOBO Co., Ltd., *t* = 50 μm), was chosen. The surfaces of the PET and PEN films (50 mm × 50 mm) were hydrophilized by a UV-ozone cleaner (OCA-150L-D, EYE GRAPHICS Co., Ltd.) for 10 min. The resulting flexible film was placed in the film deposition unit shown in Fig. 3 under atmospheric conditions at room temperature. The ITO NP deposition was carried out following the same procedure as for the deposition on a glass substrate. The ITO thin films on the flexible films were sealed in a polyethylene bag filled with N_2_ gas and irradiated with UV light for 5 min by a UV-ozone cleaner. The resulting ITO thin films were heat treated in air for 1 h at 100 °C and in H_2_-Ar (4% H_2_) for 1 h at 100 or 150 °C. The thickness of the ITO NP-based thin films was tuned to 300 nm by controlling the deposition time.

### Preparation of P1-based thin films on glass substrates by an ink coating method

A powder of **P1** was dispersed in ethylene glycol (5 wt%) by a homogenizer (VCX-750, Sonics & Materials, Inc., 115 W, Amp.: 20 μm) to obtain an ethylene glycol dispersion of **P1**. The dispersion was coated on glass substrates (CORNING, EAGLE XG Slim, thickness: 0.7 mm, alkaline earth boroaluminosilicate) by a bar coater (No. 6) at 10 °C, and the resulting **P1**-based thin films were heat treated in air for 1 h at 150 °C and in Ar-H_2_ (4% H_2_) for 1 h at 150, 200, 300, 400, or 500 °C. The thickness of the resulting thin films was adjusted to 300 nm by controlling the coating times.

### Fabrication of P1-based thin films on glass substrates by a spray coating method

A water dispersion (5 wt%) of **P1** was coated on hydrophilized glass substrates. A pressure sprayer (Dahlia #2260-2-L Hand Pressure Sprayer) was used for the spray coating. Before the coating, the glass substrate was inclined 45° to the horizontal plane and cooled to 10 °C. The thickness of the resulting spray-coated thin films was adjusted to 300 nm by controlling the coating times. The resulting ITO thin films were heat treated in air for 1 h at 150 °C and in Ar-H_2_ (4% H_2_) for 1 h at 150, 200, 300, 400, or 500 °C.

### Preparation of P1-based thin films on glass substrates by a spin coating method

A hydrophilized glass substrate was put on a spin coater (MS-B150, Mikasa Corporation) at room temperature. Then, a water dispersion (5 wt%) of **P1** was cast on the substrate and spin coated at 2000 rpm for 2 min. The thickness of the ITO thin film was controlled by the spin coating times. After heat treatment of the **P1**-based thin films on the glass substrates in air for 1 h at 150 °C and in Ar-H_2_ (4% H_2_) for 1 h at 150, 200, 300, 400, or 500 °C, the resistivity of the films was measured.

### Fabrication of P1-based thin films on glass substrates by drop casting

Initially, a hydrophilized glass substrate was inclined 45° to the horizontal plane and cooled to 10 °C. Then, a water dispersion (5 wt%) of **P1** was dropped on the substrate by a pipet. Through repeated casting, the thickness of the resulting spray-coated thin film was adjusted to 300 nm. The resulting ITO thin films were heat treated in air for 1 h at 150 °C and in Ar-H_2_ (4% H_2_) for 1 h at 150, 200, 300, 400, or 500 °C.

### SAXS measurements of the P1- and C1-based water droplets

The mean particle size in the water droplets of the NP-mists was determined by SAXS measurements at the SPring-8 BL19B2 beamline equipped with a PILATUS 2 M detector (Dectoris). The camera length was 3046 mm, and the wavelength of the beam was 0.0689 nm (18.0 keV). The **P1**-, **P2**-, and **C1**-based water droplets in the corresponding mist, generated by the mist generation unit shown in Fig. 3, were continuously introduced into a custom-made measurement cell with a path length of 5 mm. The scattering profiles were characterized by Rigaku SmartLab Studio II MRSAXS software.

### Characterization equipment

Unless otherwise noted, all the characterization tests were performed under atmospheric conditions based on our previous studies^5,37,41^. The average temperature was 28 °C. The crystallographic structures of the ITO powders were identified by a Rigaku Ultima-IV XRD measurement system using a Cu Kα radiation source (40 kV, 40 mA) equipped with a D/teX Ultra detector (the Bragg–Brentano geometry; 2*θ*/*θ* scan; step: 0.02°; speed: 5.0°/min; a focal beam condition). Silicon standard reference material (NIST: 640f.) was used for the calibration. The crystallite size of the ITO NPs was calculated by Scherrer’s equation^46^ (Scherrer constant: 1.33) using the half width of the (222) diffraction peak. The size and shape of the as-prepared particles were observed by TEM using a Hitachi H7650 system with an acceleration voltage of 100 kV. HR-TEM images were taken by a JEM-2100F (JEOL Co., Ltd.) HAADF-STEM images were obtained by an FEI TITAN 80-300 instrument at 200 kV equipped with an EDS analyzer. The molar ratios of Sn/In in the as-obtained powders were calculated from the results of ICP-AES measurements, carried out using a Perkin Elmer Optima 3300XL. Resistivity measurements of the ITO NP powder compacts were conducted based on a 4-point technique with application of a pressure of 20 kN using a Loresta-GP with an MCP-PD51 powder resistance measurement system (Mitsubishi Chemical Analytech Co., Ltd.). The size of the green compacts was 20 mm in diameter and approximately 1 mm in thickness. The measurements were carried out five times before and after annealing in air at 300 °C for 30 min and sequentially in a 4% H_2_/Ar atmosphere at 300 °C for 30 min. The average value of the five measurements was applied as the resistivity. The film thickness was measured at 6 different points by a stylus-type step profiler, and the average thickness was used for characterization. The sheet resistance obtained by a 4-point technique on an ACCENT HL5500PC system was used to calculate the resistivity of the ITO thin films on glass substrates. No difference was found between the resistivities of the films on boroaluminosilicate and on pure silica substrates. Haziness of the ITO thin films were determined by a SH7000 haze meter (Nippon Denshoku Industries Co., LTD). The measurements were carried out based on ISO 14,782. Bending tests of the ITO thin films on flexible substrates were carried out by a U-shaped folding tester (DMLHB-FS-C, YUASA SYSTEM Co., Ltd.). The absorbance and transmittance of the ITO films were measured by a UV–Vis–NIR spectrophotometer (V-670 JASCO Corporation). The roughness of the ITO thin films was evaluated by a JEOL JSPM-5200 atomic force microscope.

## Conclusions

In the present study, we designed and synthesized protrusion-rich ITO NPs with high dispersion stability in water, without using any surfactants, that are applicable for the NP-mist deposition method, and the deposited ITO thin films exhibited remarkably low resistivity, high transparency in the visible light region, and high mechanical durability after annealing at 150 °C for 1 h under a 4% H_2_ atmosphere. The properties enabled us to fabricate ITO thin films on not only glass substrates but also flexible films, such as PET and PEN. Comparison investigations using cubic-shaped ITO NPs with low dispersion stability in water revealed that utilization of the highly water dispersible protrusion-rich ITO NPs played a critical role in fabricating high-performance ITO thin films. In particular, *in-situ* SAXS measurements clarified that the protrusion-rich ITO NPs continuously deposited on substrates in a primary particle state from the droplets of the mist to form densely packed ITO thin films. Our ITO NP-based thin films obtained by the NP-mist deposition method have the following advantages: (i) the single-crystalline protrusion-rich ITO NPs were readily dispersed in water for a long period without the use of surfactants or dispersants; (ii) the ITO NP-based thin films can be manufactured under mild atmospheric conditions without the use of expensive vacuum production systems or harmful and environmentally undesirable chemicals that are used in the current sputtering and etching procedure; (iii) low resistivity, high transparency, and high mechanical durability can be achieved with low temperature annealing applicable for the deposition of ITO thin films on flexible substrates; and iv) by designing the shape of the deposition nozzle in the deposition unit of the NP-mist deposition system, ITO thin films can be uniformly fabricated on not only flat but also curved substrates. The current NP-mist deposition method might become a promising and powerful technique for the production of key materials essential for sustainable progress in PE technology as well as next-generation on-demand fabrication processes for wearable devices.

## Supplementary Information

Supplementary Information.
