# Supplementary material for: A nanoparticle-mist deposition method: fabrication of high-performance ITO flexible thin films under atmospheric conditions
Source: Sci Rep. 2021 May 19;11:10584. doi: 10.1038/s41598-021-90028-6 (PMC8134459; doi:10.1038/s41598-021-90028-6)
Supplement: Supplementary file 1 — Supplementary Information. [file 41598_2021_90028_MOESM1_ESM.pdf]

A Nanoparticle-Mist Deposition Method: Fabrication of High-Performance ITO Flexible Thin Films under Atmospheric Conditions

Ryoko Suzuki[1,2], Yasutaka Nishi[1,2], Masaki Matsubara[1,3], Atsushi Muramatsu[1], and Kiyoshi Kanie[1,*]

[1]Tohoku University, Institute of Multidisciplinary Research for Advanced Materials, Sendai, 980-8577, Japan

[2] Nikon Corporation, 10-1, Asamizodai, 1-chome, Minami-ku, Sagamihara-city, 252-0328, Japan

[3] National Institute of Technology, Sendai College, 48 Nodayama, Medeshima-Shiote, Natori 981-1239, Japan

*Correspondence to kanie@tohoku.ac.jp

Supplementary Information

1. AFM observations of the surfaces of the ITO thin films on glass substrates

Figure S1 exhibits AFM images of the **P1** thin films on glass substrates prepared by the (a) mist deposition, (b) spray coating, (c) bar coating, (d) spin coating, and (e) drop casting methods. The values of the average roughness of the films determined by the AFM observations were also shown in the images.

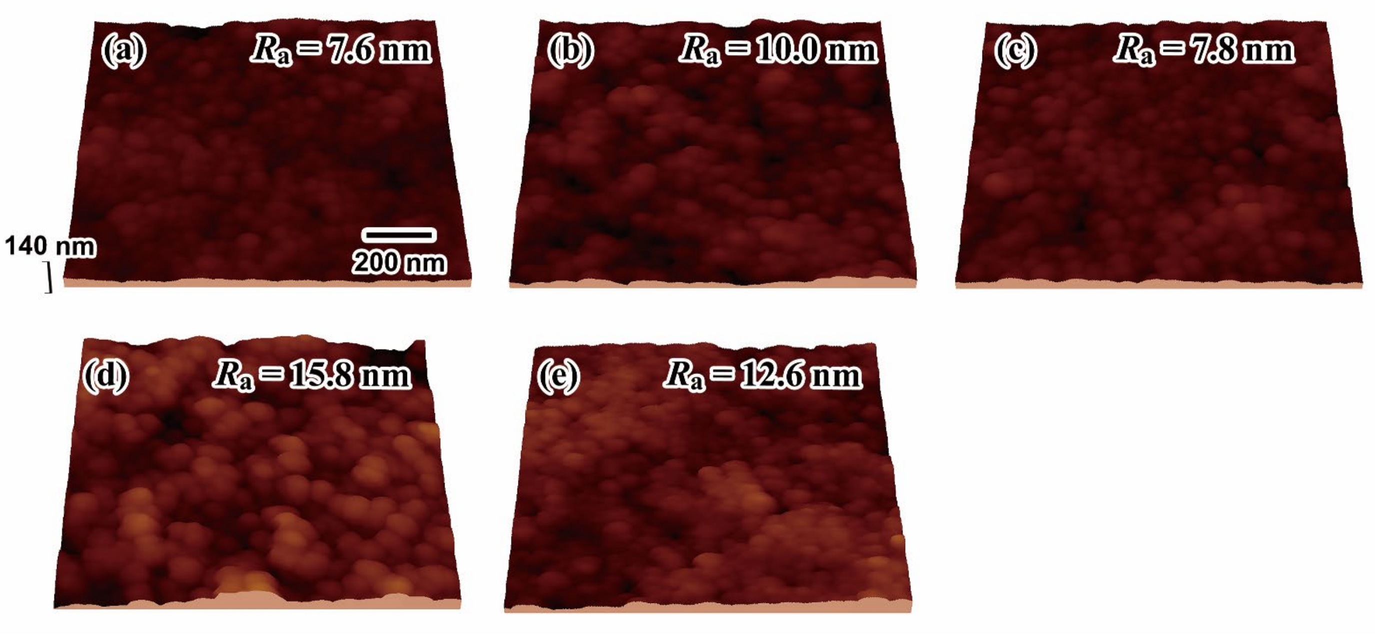

Figure S1. The results of AFM observations of the **P1** thin films on glass substrates fabricated by the (a) mist deposition, (b) spray coating, (c) bar coating, (d) spin coating, and (e) drop casting methods. The scale bar shown in (a) is common to all images. The average roughness of the films (R_a) calculated from the AFM images were also shown in the images.

Figure S2 shows AFM images of the **C1** and **R1**-based ITO thin films on glass substrates prepared by the NP-mist deposition method after annealed at 150 °C.

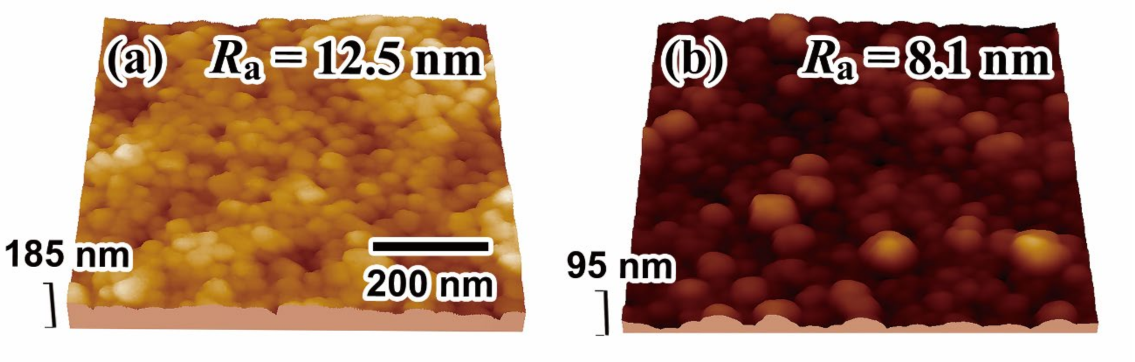

Figure S2. AFM images of the (a) **C1** and (b) **R1**-based ITO thin films on glass substrates prepared by the NP-mist deposition method after annealed at 150 °C. The scale bar shown in (a) is common to (b). The average surface roughness of the films calculated from the AFM images (R_a) were also shown in the images.

2. Characterization of the results of SAXS measurements

Figure S3 summarizes the results of characterization of SAXS profiles of (a) **C1**-, (b) **P1**-, and (c) **P2**-based water droplets of the NP-mists.

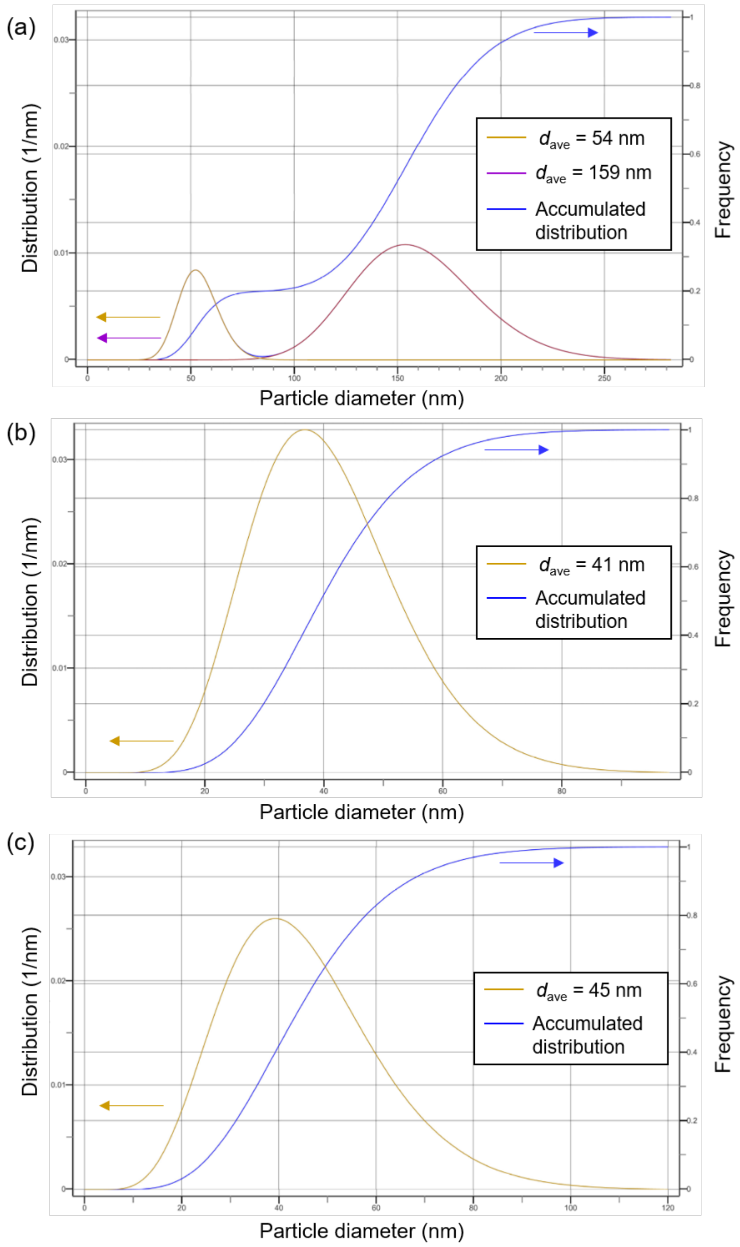

Figure S3. Results of characterization of the SAXS profiles of (a) **C1**-, (b) **P1**-, and (c) **P2**-based water droplets of the NP-mists. The scattering profiles were characterized by Rigaku SmartLab Studio II MRSAXS software.
